# Online Deglycosylation of Monomeric Intact Proteins Using the PNGase Rc Immobilized-Enzyme Reactor

**DOI:** 10.1021/acsomega.6c00577

**Published:** 2026-04-21

**Authors:** Katarína Molnárová, Petr Novák, Jana Nováková, Petr Pompach

**Affiliations:** † AffiPro s.r.o., Nad Safinou II 365, Vestec 252 50, Czech Republic; ‡ Institute of Microbiology of the Czech Academy of Sciences, Videnska 1083, 142 20 Prague, Czech Republic

## Abstract

Protein glycosylation is essential, as variations in glycan structures can influence the structural stability, biological activity, and therapeutic performance of proteins. The complexity of glycoproteins typically requires advanced analytical approaches for structural determination. Removal of glycans from glycoproteins simplifies the analysis of proteins and enables characterization of glycosylation sites and polypeptide structures. In this study, we evaluated the performance of an immobilized enzyme reactor (IMER) containing peptide *N*-glycosidase from *Rudaea cellulosilytica* (PNGase Rc) for online deglycosylation of monomeric intact glycoproteins. We selected human haptoglobin, human transferrin, *anti*-hHD6, bevacizumab, and trastuzumab as model substrates. The results demonstrate that the PNGase Rc IMER enables rapid and efficient online deglycosylation of glycoproteins and antibodies bearing complex sialylated and core-fucosylated glycans, highlighting its potential in bioanalytical workflows, quality control during biopharmaceutical development, and stability tests.

## Graphical abstract

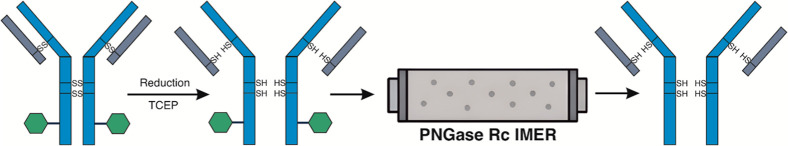

## Introduction

Protein glycosylation, in which monosaccharide units are attached to a protein backbone through enzymatic pathways, is involved in many important biochemical processes and is considered one of the most important post-translational modifications.^1^ It affects not only the stability and secretion of proteins, cell–cell interactions, and receptor–ligand recognition mechanisms but also the biological activity, plasma half-life, immunogenicity, pharmacodynamics, and pharmacokinetics of biotherapeutics.^2–5^ Therapeutical monoclonal antibodies (mAbs) are among the best known pharmaceuticals that are based on glycoproteins.^6^ Over 90% of the mAbs are derived from immunoglobulin G (IgG), in which *N*-glycosylation occurs at Asn297 of the Fc region and is responsible for modulating immune responses and interactions with other proteins.^7,8^ In cancer treatment, mAbs are used to increase the response of the immune system against the tumor cell, thus inhibiting the cancerous cell growth and helping with the destruction and elimination.^9^ Among the most commonly used mAbs are trastuzumab, bevacizumab, cetuximab, or margetuximab, which are used as an additional treatment for breast, colorectal, prostate, or lung cancer.^10^

Mass spectrometry (MS)-based methods have become a method of choice in the analysis of mAbs that can provide information on sequence, post-translational modifications, and glycosylation profiles.^11^ The enzymatic removal of the attached glycans from the protein backbone can reduce the complexity of the MS analysis, which can lead to a better understanding of the protein structure and modifications. In glycoproteomics, peptide-*N*-glycosidase F (PNGase F) is most used for glycan removal. It cleaves the linkage between the Asn residue and the *N*-acetylglucosamine (GlcNAc).^12^ Jmeian et al. developed a monolithic PNGase F enzyme reactor fabricated in fused silica using a glycidyl methacrylate-*co*-ethylene dimethacrylate polymer and integrated it into an LC–MS workflow. The authors demonstrated the applicability of this system for the characterization of released neutral and acidic *N*-glycans by LC–MS analysis.^13^ In another study, oriented immobilization of PNGase F onto a methacrylate-based monolithic support via glutathione-*S*-transferase–glutathione affinity coupling was employed to release *N*-glycans from denatured ribonuclease B and fetuin, as well as from native IgG. The released glycans and the corresponding deglycosylated proteins were subsequently analyzed by MALDI-MS.^14^

Gramlich et al. showed the potential of PNGase Rc for *N*-glycan hydrolysis from both tryptic peptides and native glycoproteins at a low pH. The favorable properties of PNGase Rc, including activity at acidic pH and compatibility with reducing agents such as *tris*(2-carboxyethyl)­phosphine hydrochloride (TCEP) and denaturants like urea, make this enzyme well suited for online intact protein deglycosylation workflows.^15,16^

In this report, we demonstrate the potential of the PNGase Rc IMER for rapid online deglycosylation of reduced monomeric glycoproteins, including human haptoglobin (Hp), human transferrin, *anti*-hHD6, and the therapeutic monoclonal antibodies trastuzumab and bevacizumab. The column retained its activity over more than 150 consecutive measurements, highlighting its robustness, suitability for repeated use in bioanalytical workflows, and applicability to pharmaceutical research and development.

## Experimental Section

### Chemicals and Materials

Bevacizumab was supplied by Roche Pharma AG (Grenzach-Wyhlen, Germany), and trastuzumab was supplied by Biotec Services International Ltd. (Bridgend, United Kingdom). The *anti*-hHD6 antibody was prepared by the Laboratory of Structural Biology, Institute of Biotechnology, Czech Academy of Sciences. Human haptoglobin (phenotype 2–1), human transferrin, ammonium bicarbonate, *tris*(2-carboxyethyl)­phosphine (TCEP), acetonitrile, water, propan-2-ol, and formic acid (all LC–MS grade) were purchased from Merck (Darmstadt, Germany).

### Instrumentation and Experimental Conditions

Automated sample handling and online deglycosylation were carried out using the HDX workstation (AffiPro, Czech Republic). The scheme of the system is shown on Figure 1. Samples of 50 mM ammonium bicarbonate buffer (pH 8.2) and 40 mM TCEP were placed in a sample tray at 4 °C. Ten microliters of the protein sample at a concentration of 0.03 mg/mL (0.3 μg per injection) were added to 40 μL of 50 mM ammonium bicarbonate and then transferred to 50 μL of 40 mM TCEP, pH 2.3. The disulfide bond reduction was carried at 40 °C for 5 min. The automated sample handling system injected 100 μL of the reaction mixture into the sample loop and switched the injection valve. Online deglycosylation was performed at room temperature using a PNGase Rc IMER (2.1 × 20 mm, AffiPro, Czech Republic) at a flow rate of 50 μL/min, followed by protein desalting on an OPTI-TRAP (0.5 × 2 mm) trap column (Optimize Technologies Inc., Oregon City, Oregon, USA) heated to 55 °C for 10 min with 0.2% formic acid delivered by an Agilent 1260 Infinity III isocratic pump (Agilent Technologies, Santa Clara, California, USA). By switching the trap valve, the protein was eluted by an acetonitrile gradient [(min)/% B] 0/10-9/10-16/70-16.5/90-17/90-18/10-23/10] delivered by the Agilent 1290 Infinity II HPLC system (Agilent Technologies, Santa Clara, California, USA) at a flow rate of 20 μL/min. The mobile phase A consisted of 5% acetonitrile with 1% propan-2-ol and 0.1% formic acid, and the mobile phase B consisted of 95% acetonitrile with 1% propan-2-ol and 0.1% formic acid. The maXis Q-TOF mass spectrometer (Bruker Daltonics, Bremen, Germany) was operated in the survey MS mode in a mass range of *m*/z 50–3000. The acquired data were analyzed and processed by DataAnalysis 4.4 (Bruker Daltonics) and deconvoluted by UniDec^17^ with the following parameters: data processing range, *m*/z 500–3000; charge range, 1–80; allowed mass range, 1,000–80,000; sampling rate, 10 Da; peak detection range, 500 Da; and peak detection threshold, 0.1.

**Figure 1:**
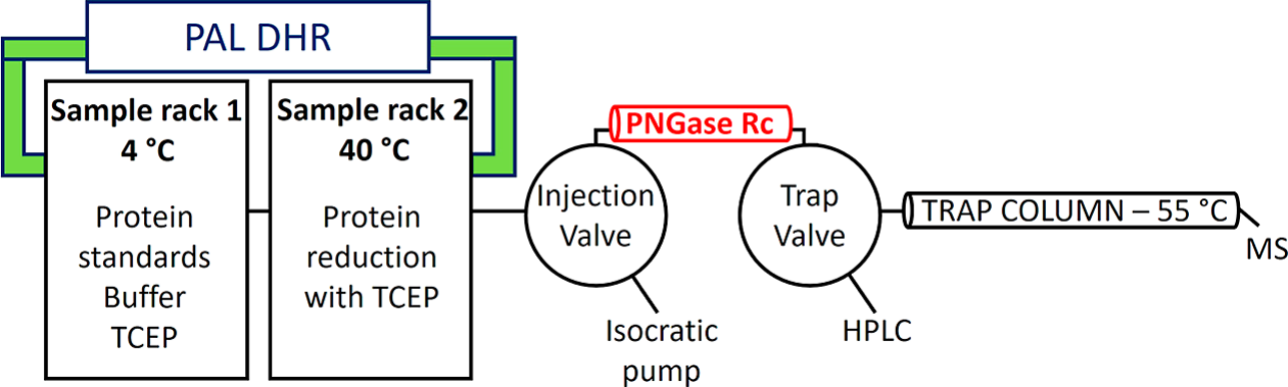
Schematic of the automated sample handling system for online protein deglycosylation and desalting. Protein samples, along with 50 mM ammonium bicarbonate and 40 mM TCEP stock solutions were placed in a sample tray chilled to 4 °C, while a second tray heated to 40 °C was used for disulfide bond reduction with TCEP buffer.

## Results and Discussion

The performance of the PNGase Rc IMER for the removal of complex *N*-glycans from intact proteins was tested using heavily glycosylated human Hp with four glycosylation sites (Asn184, Asn207, Asn211, and Asn241)^18^ located at the β-chain and occupied by complex *N*-glycans terminated with sialic acid. Haptoglobin phenotype 2-1, used in this study, forms multimers consisting of β-chain and disulfide linked α1 and α2 chains.^19^ The detection of glycosylated Hp β-chain requires reduction of the disulfide bonds by incubating the protein with a reducing reagent. TCEP is a common reducing reagent used for fast reduction of disulfide bonds at various pH levels. Forty degrees Celsius, the maximum temperature of the PAL tray, and 5 min of incubation were sufficient for complete reduction of Hp disulfide bonds. Individual signals corresponding to the glycosylated β-chain were observed in the *m*/*z* range of 35,800–37,400 (Figure 2a). The limited resolution of the mass spectrometer did not allow assigning the different glycoforms of the haptoglobin β-chain, which is occupied by biantennary, triantennary, or tetraantennary glycans with various amounts of sialic acid and fucose.^20,21^ The nonglycosylated α1 and α2 chains were observed at *m*/*z* 9190 and 15,940. When the PNGase Rc IMER was integrated into the flow system, a signal at *m*/*z* 27,270 was detected, demonstrating complete deglycosylation of the haptoglobin β-chain (Figure 2b).

**Figure 2:**
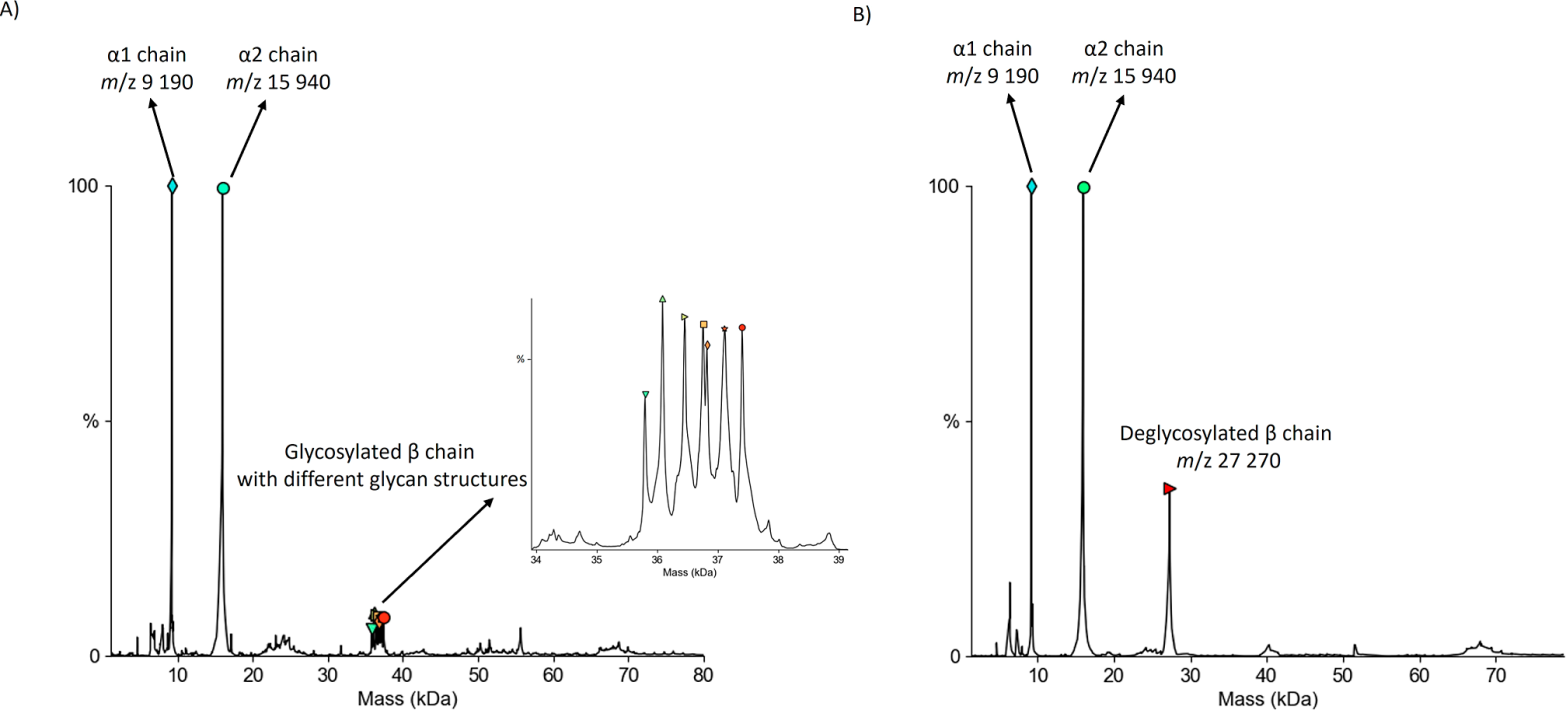
(A) Mass spectrum of human Hp without the PNGase Rc IMER implemented in the system. The base peak signals of the spectrum correspond to both α1 and α2 chains. The signal of the β chain is spread across its different glycoforms. (B) Mass spectrum of human Hp with the PNGase Rc IMER implemented in the system. The signal of the deglycosylated β chain is higher compared to its glycosylated form and observed only at *m*/*z* 27,270. This indicates complete removal of glycans from the Hp β chain.

Human transferrin is glycosylated at positions Asn432 and Asn630 by sialylated biantennary glycans.^22^ The signal corresponding to the glycosylated reduced monomeric protein was detected at *m*/*z* 79,540 (Figure 1A). After deglycosylation, a signal at *m*/*z* 75,125 was observed (Figure 1B), indicating complete deglycosylation of the reduced protein.

*Anti*-hHD6 was selected as a model antibody to investigate the ability of the PNGase Rc IMER to deglycosylate mAbs, thereby assessing its performance on therapeutic antibodies. This antibody, derived from IgG, consists of two light chains and two glycosylated heavy chains. For the light chain, a single peak at *m*/z 23,950 (Figure 3a) was observed, which remained unchanged after the deglycosylation with PNGase Rc IMER (Figure 3b), confirming that no glycosylation is present. In contrast, for the heavy chain, two peaks were detected without the enzyme column (Figure 3a) at *m*/z 49,960 and 50,120, representing core-fucosylated biantennary glycans either without or with galactose, respectively. Following online deglycosylation on the PNGase Rc column (Figure 3b), we obtained a single sharp peak at *m*/z 48,510 corresponding to a completely deglycosylated heavy chain.

**Figure 3:**
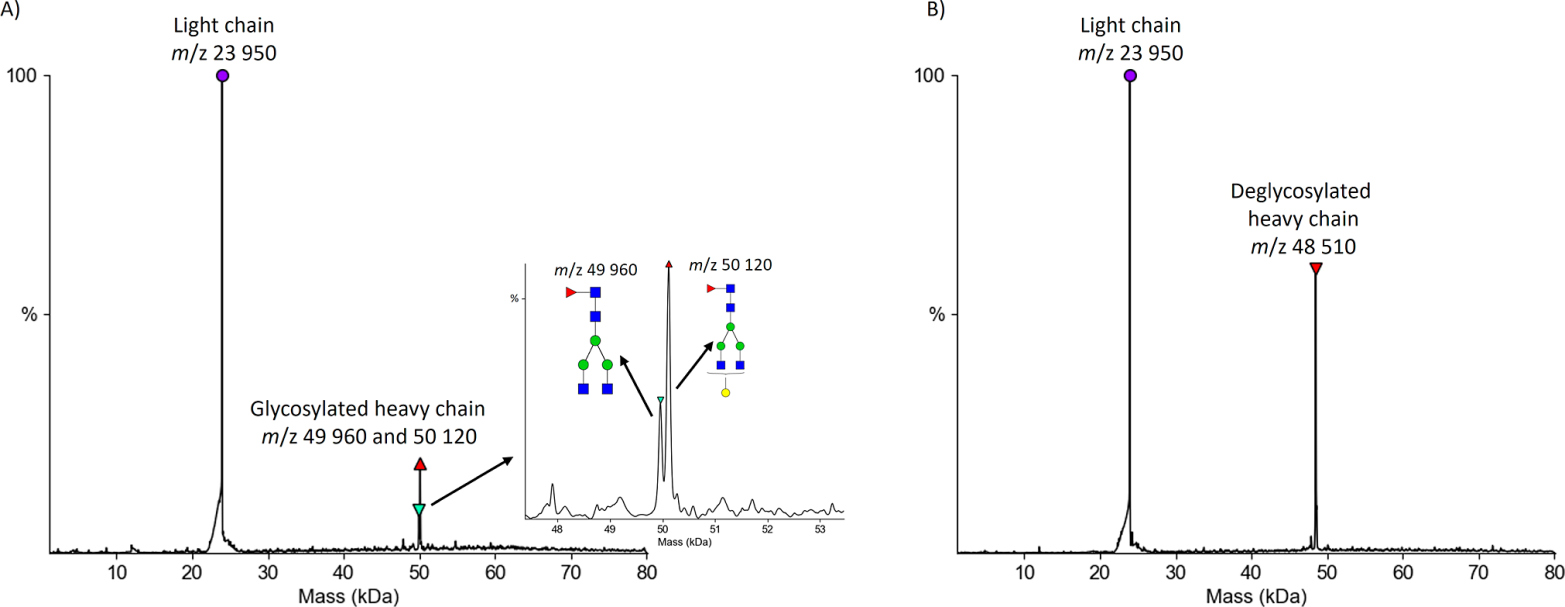
(A) Deconvoluted spectra of *anti*-hHD6 obtained without the PNGase Rc IMER. (B) Deconvoluted spectra of *anti*-hHD6 obtained with the PNGase Rc IMER.

The potential of PNGase Rc IMER to remove *N*-linked glycans from therapeutic mAbs was demonstrated by using bevacizumab and trastuzumab. Most therapeutic mAbs are derived from IgG with a glycosylation site at Asn297.^7^ For bevacizumab and trastuzumab, the humanized mAb IgG1 is produced in Chinese hamster ovary and consists of two light and two glycosylated heavy chains.^23,24^ According to the National Center for Biotechnology Information^25^ and the European Medicines Agency,^26^ the molecular weight of bevacizumab is approximately 149,000 Da, with the light chain consisting of 214 amino acids (approximately 23,000 Da) and the heavy chain with 453 residues (approximately 49,000 Da without the glycan moiety), respectively. The mass spectrum of bevacizumab without treatment by PNGase Rc is shown in Figure 4a. The signal corresponding to the light chain was observed at *m*/z 23,450, and the heavy chain bearing two different glycoforms was observed at *m*/z 51,160 and at *m*/*z* 51,320. The mass of 51,160 Da corresponds to the agalactosylated glycoform as a major glycoform,^27^ and a mass of 51,320 Da was matched to the monogalactosylated glycoform, each core-fucosylated. Online deglycosylation (Figure 4b) resulted in no change in the light chain signal, whereas a new peak at *m*/*z* 49,720 was detected, corresponding to the deglycosylated heavy chain.

**Figure 4:**
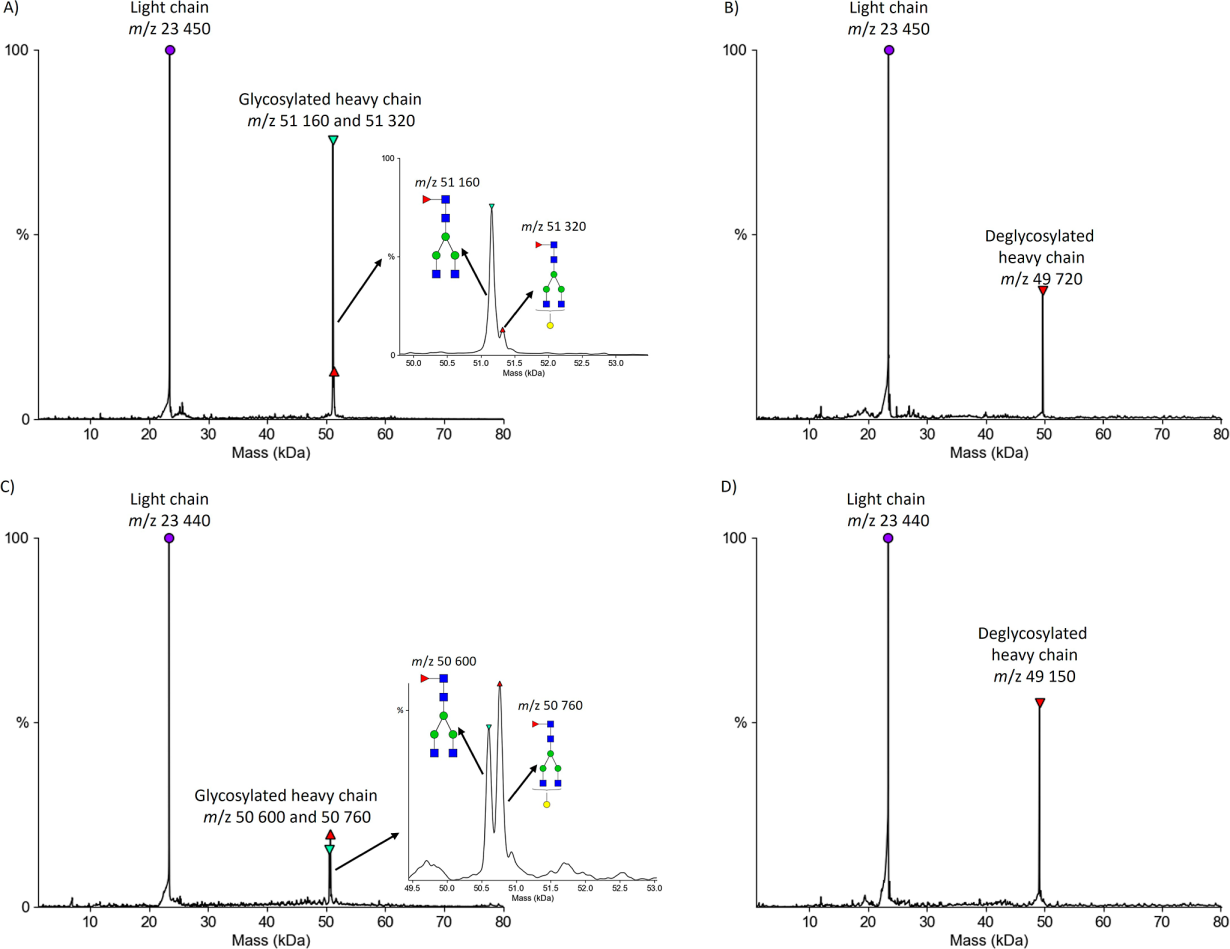
(A) Mass spectrum of bevacizumab showing two glycoforms of the heavy chain at *m*/*z* 51,160 and *m*/*z* 51,320. (B) Mass spectrum of bevacizumab after deglycosylation using the PNGase Rc IMER, showing the completely deglycosylated heavy chain at *m*/*z* 49,720. (C) Mass spectrum of trastuzumab showing glycosylated heavy chain at *m*/*z* 50,600 and *m*/*z* 50,760. D) Mass spectrum of trastuzumab after PNGase Rc IMER deglycosylation showing a single signal of the heavy chain at *m*/*z* 49,150.

For trastuzumab, the light chain was detected at *m*/z 23,440, and the glycosylated heavy chain carrying core-fucosylated biantennary glycans without or with galactose was detected at *m*/z 50,600 and 50,760, respectively (Figure 4c). After the online removal of the attached glycans from the heavy chain (Figure 4d), only a signal at *m*/z 49,150 was detected, representing the fully deglycosylated heavy chain and confirming the efficiency of the PNGase Rc column.

In a previous study by Krenkova et al., the PNGase F IMER was used for deglycosylation of IgG, RNase B, and bovine fetuin with follow-up mass spectrometry detection of both released glycans and intact proteins using MALDI-TOF MS.^14^ The authors demonstrated an effective method for rapid protein deglycosylation, which, however, requires additional sample handling prior to mass spectrometry measurement. The other study by Jmeian et al., which focused on released *N*-glycans, implemented a PNGase F IMER with online LC–MS detection.^13^ The authors show the effectiveness of the method by identifying many different glycans released from a single protein or from a complex plasma mixture. Our approach is not directly intended for released *N*-glycan analysis; instead, it demonstrates the ability to measure intact proteins after rapid online deglycosylation using PNGase Rc at low pH. Samples prepared immediately prior to mass spectrometry analysis using the automated sample-handling platform can reduce variability associated with autosampler residence time, as some proteins may undergo degradation or precipitation during storage in the autosampler.

Although all glycoproteins in this study were effectively deglycosylated, the method’s efficiency depends on the characteristics of the individual glycoprotein. Therefore, optimizing method parameters—including the isocratic flow rate, glycoprotein incubation time with TCEP, and/or the addition of denaturing reagent—may improve the overall efficiency of the deglycosylation process.

The PNGase Rc IMER stability over a series of injections was tested by using the trastuzumab antibody. The measurements were carried out with the enzyme column kept at the laboratory temperature. As shown in Figure 5, the results indicate that the column performance remained stable over the course of the 150 measurements, and no glycosylated form of the trastuzumab antibody heavy chain was detected. The activity of PNGase Rc in the presence of 5% acetonitrile and 5% propan-2-ol was evaluated using a bottom-up approach with human 2–1 haptoglobin, which was online-digested by a coimmobilized nepenthesin-2/pepsin IMER. In the absence of an organic solvent, the peptide IEKVVLHPNYSQVD remained fully deglycosylated throughout 30 injections. In contrast, in the presence of organic solvent, a marked loss of enzymatic activity was observed after only a few injections (Figure 2).

**Figure 5:**
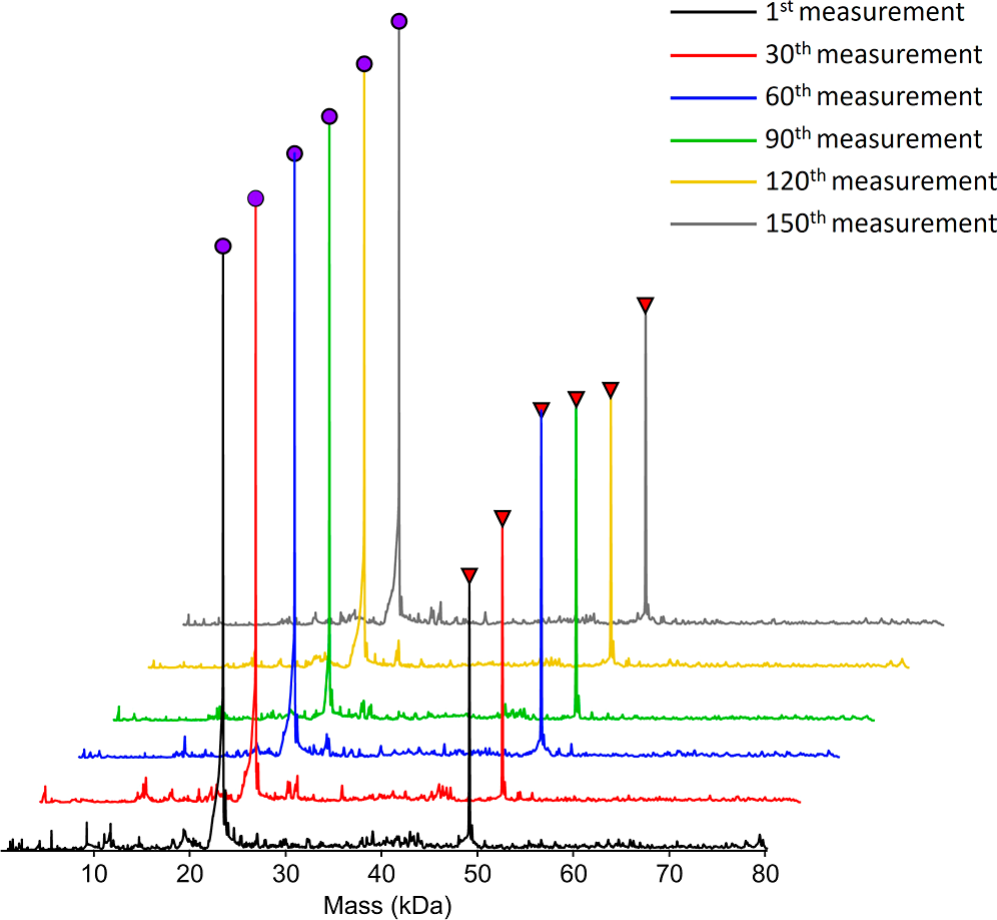
Monitoring the stability and performance of PNGase Rc IMER. Trastuzumab heavy chain was completely deglycosylated after 150 injections.

## Conclusions

Here, we demonstrate the potential of the PNGase Rc IMER for the online removal of *N*-glycans from intact monomeric glycoproteins reduced with TCEP. The column showed high sensitivity toward Hp, which has multiple glycosylation sites with various glycan structures, confirming its suitability for the profiling of complex glycoproteins. Bevacizumab and trastuzumab exhibited similar glycosylation profiles, with core-fucosylated biantennary glycans being the most abundant. Online deglycosylation with the PNGase Rc column resulted in a complete removal of the glycans from the heavy chains of both antibodies, proving that the column can be used for the rapid online deglycosylation of intact therapeutic mAbs. Moreover, the column remained stable over 150 measurements without any loss of activity, even when it was maintained at laboratory temperature. The significantly reduced reaction time, together with full automation and the absence of manual handling steps, substantially improves analytical throughput compared to conventional off-line deglycosylation workflows. These findings highlight the potential of the PNGase Rc IMER in the development of glycoprotein-based biopharmaceuticals, where detailed structural characterization of the protein part is critical for quality control, monitoring batch-to-batch variability, and stability testing.

## Supplementary Material



## Notes

Mass spectrum of human transferrin, chromatograms, and PNGase Rc activity test in the presence of organic solvent (PDF)

## Abbreviations

GlcNAc: *N*-acetylglucosamine
HDX-MS: hydrogen–deuterium exchange mass spectrometry
Hp: haptoglobin
IgG: immunoglobulin G
IMER: immobilized-enzyme reactor
mAb: monoclonal antibody
PNGase F: peptide-*N*-glycosidase F
PNGase Rc: peptide-*N*-glycosidase from *Rudaea cellulosilytica*
TCEP: *tris*(2-carboxyethyl)­phosphine hydrochloride
