# Supplementary material for: Online Deglycosylation of Monomeric Intact Proteins Using the PNGase Rc Immobilized-Enzyme Reactor
Source: ACS Omega. 2026 Apr 21;11(17):25724–9. doi: 10.1021/acsomega.6c00577 (PMC13150606; doi:10.1021/acsomega.6c00577)
Supplement: Supplementary file 1 [file ao6c00577_si_001.pdf]

Supplemental material

Online deglycosylation of monomeric intact proteins proteins using PNGase Rc immobilized-enzyme reactor

Katarína Molnárová[1]*, Petr Novák*[2]*, Jana Nováková*[1]*, Petr Pompach*[1*]

1. AffiPro s.r.o., Nad Safinou II 365, Vestec 252 50, Czech Republic

2. Institute of Microbiology of the Czech Academy of Sciences, Videnska 1083, 142 20, Prague, Czech Republic

Supplemental Figure 1

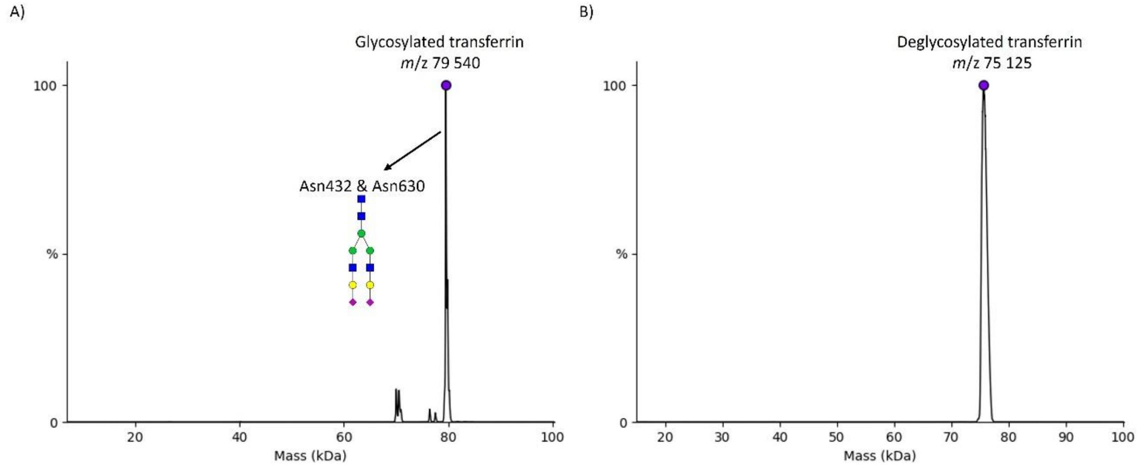

Supplemental Figure 1. A) Mass spectrum of human transferrin without the PNGase Rc IMER implemented in the system. B) Mass spectrum of human transferrin with the PNGase Rc IMER implemented in the system. The shift in mass between glycosylated and deglycosylated transferrin corresponds to loss of two bi-antennary doubly sialylated glycans.

Supplemental Figure 2

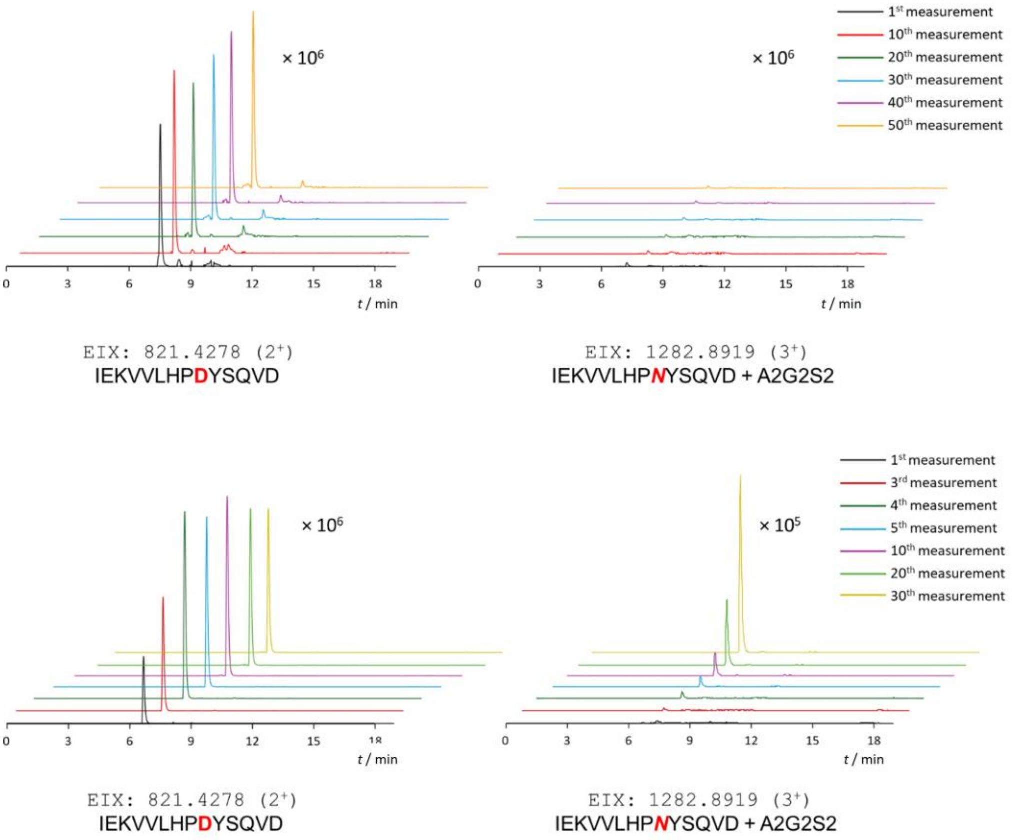

Supplemental Figure 2: Upper chromatograms showing XIC of deglycosylated peptide IEKVVLHPDYSQVD (left) and glycosylated peptide IEKVVLHPNYSQVD + A2G2S2 in the absence of organic solvent. Lower chromatograms represent of deglycosylated peptide IEKVVLHPDYSQVD (left) and glycosylated peptide IEKVVLHPNYSQVD + A2G2S2 in the presence of organic solvent. The glycosylation activity was lost after several injection of organic solvent on the PNGase Rc IMER. A2G2S2 represents biantennary glycan with two terminal sialic acids.

Supplemental Methods

PNGase Rc activity test in the presence of organic solvent.

Human 2-1 haptoglobin (100 pmol) was injected by robotic system on the co-immobilized Nepenthesin-2/pepsin IMER (2.1 x 20mm, AffiPro, Czech Republic) connected to the PNGase Rc IMER. After each injection of the protein, 100µL of 5% acetonitrile and 5% 2-propanol was injected on the columns. Online digestion and deglycosylation was performed at 4°C at a flow rate of 200 µL/min, followed by peptide desalting on trap column (SecurityGuard ULTRA cartridges, Polar C18 2.1mm, Phenomenex) for 3 minutes with 0.4% formic acid delivered by an Agilent 1260 Infinity III binary pump (Agilent Technologies, Santa Clara, California, USA). By switching the trap valve, the peptides were separated by analytical column (Luna Omega 1.6 µm Polar C18, 100 x 1mm, Phenomenex) and eluted by acetonitrile gradient [(min)/% B] 0/5-5/10-50/35-53/99-54/5-60/5] delivered by Agilent 1290 Infinity II HPLC system (Agilent Technologies, Santa Clara, California, USA) at a flow rate of 50 µL/min. The mobile phase A consisted of 5% acetonitrile with 0.4% formic acid and mobile phase B consisted of 95% acetonitrile with 0.4% formic acid. The maXis™ Q-TOF mass spectrometer (Bruker Daltonics, Bremen, Germany) was operated in MS/MS mode in a mass range of *m*/z 50-1800. The acquired data were analyzed and processed by DataAnalysis 4.4 (Bruker Daltonics).
